# Supplementary material for: Identification of photoperiod-induced specific miRNAs in the adrenal glands of Sunite sheep (Ovis aries)
Source: Front Vet Sci. 2022 Jul 22;9:888207. doi: 10.3389/fvets.2022.888207 (PMC9354845; doi:10.3389/fvets.2022.888207)
Supplement: Supplementary file 1 [file Data_Sheet_1.ZIP › Supplementary file/miRDeep2 file.zip!/miRDeep2 file/Know_Structure/LP42A2/oar-mir-152.pdf]

miRBase precursor : oar-mir-152
Total read count : 22233
oar-miR-152 read count : 22233
remaining reads : 0

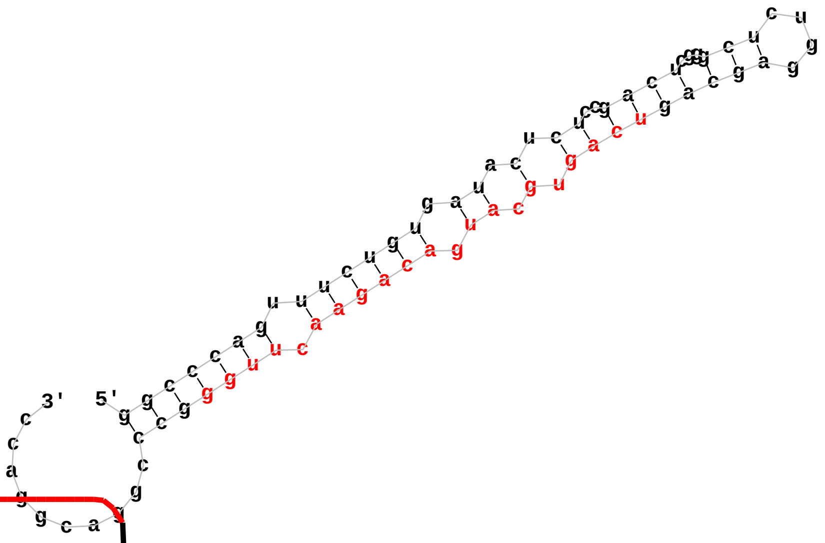

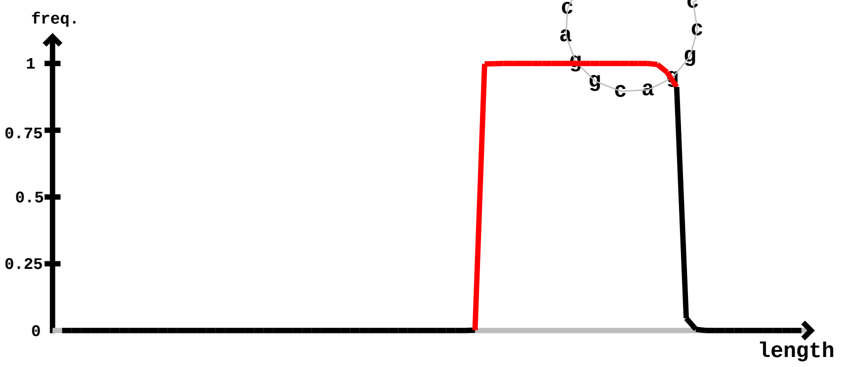

oar-miR-152

```
5'- ggcccaguuucugugauacucuccgacucgggcucuggagcagucagugcaugacagaacuugggcccggacggacc   -3'    exp
    (((((((.((((((.((.(.((..((((...(((....))))))))).).)).)))))).))))))).........    reads      mm         sample
    ..........................................gucagugcaugacagaacuug..............    6          0          seq
    ..........................................gucagugcaugacagaacuugg.............    26         0          seq
    ...........................................ucagugcaugacagaac.................    1          0          seq
    ...........................................ucagugcauCacagaac.................    2          1          seq
    ...........................................ucagugcauCacagaacu................    16         1          seq
    ...........................................ucagugcaugacagaacu................    67         0          seq
    ...........................................ucagugcauCacagaacuu...............    24         1          seq
    ...........................................ucagugcaugacagaacuu...............    617        0          seq
    ...........................................ucagugAaugacagaacuu...............    1          1          seq
    ...........................................ucagugAaugacagaacuug..............    2          1          seq
    ...........................................ucagugcaugacagaacuug..............    1209       0          seq
    ...........................................ucagugcaugacagaacuugg.............    19206      0          seq
    ...........................................ucagugcaugacagaacuuggg............    919        0          seq
    ...........................................ucagugcaugacagaacuugggc...........    99         0          seq
    ...........................................ucagugcaugacagaacuugggcc..........    2          0          seq
    ............................................cagugcaugacagaacuu...............    1          0          seq
    ............................................cagugcaugacagaacuug..............    2          0          seq
    ............................................cagugcaugacagaacuugg.............    10         0          seq
    ............................................cagugcaugacagaacuugggc...........    1          0          seq
    .............................................agugcaugacagaacuu...............    1          0          seq
    .............................................agugcaugacagaacuug..............    1          0          seq
    .............................................agugcaugacagaacuugA.............    1          1          seq
    .............................................agugcaugacagaacuugg.............    19         0          seq
```
